# *Ulmus davidiana* var. *japonica* Nakai Upregulates Eosinophils and Suppresses Th1 and Th17 Cells in the Small Intestine

**DOI:** 10.1371/journal.pone.0076716

**Published:** 2013-10-07

**Authors:** Han-Sung Lee, Min Seong Jang, Jung-Hwan Kim, Chun-Pyo Hong, Eun-Jung Lee, Eun Ji Jeun, Chan Kim, Eun-Kyung Kim, Kwang-Seong Ahn, Bo-Gie Yang, Kwang Seok Ahn, Young Pyo Jang, Kyoo-Seok Ahn, You-Me Kim, Myoung Ho Jang

**Affiliations:** 1 Division of Integrative Biosciences and Biotechnology, Pohang University of Science and Technology, Pohang, Korea; 2 Department of Pathology, College of Korean Medicine, Kyung Hee University, Seoul, Korea; 3 Division of Pharmacognosy, College of Pharmacy, Kyung Hee University, Seoul, Korea; 4 Academy of Immunology and Microbiology (AIM), Institute for Basic Science (IBS), Pohang, Korea; Cincinnati Children's Hospital Medical Center, University of Cincinnati College of Medicine, United States of America

## Abstract

The bark of *Ulmus davidiana* var. *japonica* Nakai (Ulmaceae) has been used in traditional Korean medicine for chronic inflammation in the gastrointestinal tract. Here we investigated the frequency and cytokine profile of the major immune cells in the small intestinal lamina propria (SI LP), spleen, and mesenteric lymph nodes (MLNs) of mice treated orally with *Ulmus davidiana* var. *japonica* Nakai bark water extract (UDE) to address the immunomodulatory role of this herb in intestinal homeostasis. B6 mice were given 5g/kg UDE once daily for 14 days. They were then sacrificed, and cells were isolated from the spleen, MLNs, and SI LP. The proportion of B versus T lymphocytes, CD4^+^ versus CD8^+^ T lymphocytes, Th1 and Th17 cells, and Foxp3^+^ regulatory T cells in the spleen, MLNs, and SI LP were analyzed. The frequency of antigen-presenting cells (APCs), including dendritic cells, macrophages, and eosinophils in the SI LP and the expression of costimulatory molecules on APCs were also evaluated. The numbers and frequencies of Th1 and Th17 cells in the SI LP were significantly reduced in the UDE-treated mice compared with PBS controls. In addition, the proportion of IL-4-producing eosinophils in the SI LP was significantly elevated in the UDE-treated mice compared with controls. Taken together, these data indicate that UDE up-regulates the number and frequency of SI LP eosinophils, which can down-regulate the Th1 and Th17 responses via IL-4 secretion and contribute to intestinal homeostasis.

## Introduction

The gastrointestinal (GI) tract is the entry site for many potentially pathogenic microorganisms and constantly exposed to dietary antigens and commensal microflora [1]. The enigmatic coexistence of antigens, microflora, and host tissue requires an elaborate intestinal immune system to maintain gut homeostasis. The intestinal immune system has evolved diverse strategies to orchestrate protective immunity and immune tolerance in the host [2]. The GI mucosa, especially the lamina propria (LP), which is the loose connective tissue layer underlying the intestinal epithelium, harbors various kinds of immune cells that are associated with immune regulation. Accumulating evidence indicates that in the small intestinal (SI) LP, oral tolerance is mediated by Foxp3^+^ regulatory T (Treg) cells and antigen-presenting cells (APCs), which include dendritic cells (DCs) [3] and macrophages [4]; in contrast, effector T cells, including Th17 cells, function in host defense.

In addition, the LP of the stomach and small intestine contains more eosinophils than other tissues under healthy conditions. In general, eosinophils function as effector cells in parasitic infections and allergic disorders, and are armed with cytotoxic granular proteins such as major basic protein, eosinophil cationic protein, eosinophil-derived neurotoxin, and eosinophil peroxidase [5]. However, LP resident eosinophils at steady state show several differences from those that function under pathological conditions and are speculated to serve distinct functions. Most eosinophil research to date has focused on their pathological functions in hematopoietic and pulmonary tissues. In contrast, little is known about their physiological functions in the GI tract [6].

*Ulmus davidiana* var. *japonica* Nakai, called the ‘Japanese elm’, is a deciduous broad-leaved tree extensively found in eastern Asia. The bark of this tree is used in traditional Korean medicine for dysuria, swelling, rhinitis, and inflammatory ulceration of the GI tract. It was recently reported that a glycoprotein isolated from this tree has anti-inflammatory activities via the inhibition of inducible nitric oxide synthase and cyclooxgenase-2 in lipopolysaccharide-stimulated RAW 264.7 cells, and shows protective effects in the murine Dextran Sulfate Sodium (DSS)-induced colitis model [7]. Another study showed that *Ulmus macrocarpa* Hance has a protective effect in the experimental murine colitis model induced by DSS and 2, 4, 6-trinitrobenzene sulfonic acid (TNBS) [8]. Collectively, the plants belonging to genus *Ulmus* may have anti-inflammatory effects in the gut. However, the immunological mechanisms, in particular those involving the SI LP cells, remain unclear.

Here we show that Th1 and Th17 cells are decreased in the SI LP of mice treated orally with *Ulmus davidiana* var. *japonica* Nakai bark water extract (UDE) while the SI LP eosinophil population is markedly increased. These changes may mediate the anti-inflammatory effects of UDE in the GI tract.

## Materials and Methods

### Mice

Female 6- to 8-week-old C57BL/6 and BALB/c mice were obtained from the POSTECH Biotech Center. IL-4/GFP (green fluorescent protein) BALB/c-*4get* mice (4get mice), which are transgenic mice carrying a cassette in the interleukin (IL)-4 gene locus that allows dual expression of IL-4 enhanced GFP [9], and eosinophil-deficient ΔdblGATA mice [10] were purchased from the Jackson Laboratory (Bar Harbor, ME, USA). All the mice were maintained under specific pathogen-free conditions. All animal experiments were performed under experimental protocols approved by the Ethics Review Committee for Animal Experimentation of Pohang University of Science and Technology.

### Preparation of UDE

The raw plant materials were purchased from Omniherb (Youngcheon, Korea) and were authenticated by Professor Kyoo-Seok Ahn (College of Korean Medicine, Kyung-Hee University, Korea). An extract of *Ulmus davidiana* var. *japonica* Nakai bark was prepared by decocting with distilled water (100 g/L). The decoction was filtered, lyophilized, and kept at 4 °C. The yield of the extraction was about 2.26% (w/w).

### Oral UDE administration

Mice were divided into two groups: a UDE group and a phosphate buffered saline (PBS) control group. The UDE powder was dissolved in PBS (final concentration 500 mg/mL), and 200 µL of this preparation was orally administered to each mouse in the UDE group, whereas the control group was given the same volume of PBS, once a day for 2 weeks.

### Cell preparation from SI LP, mesenteric lymph nodes (MLNs), and spleen

The SI LP cells were obtained as previously described [11,12]. Briefly, the small intestine was isolated, and after excision of the adipose tissue and Peyer’s patches, was opened longitudinally, rinsed in cold PBS, and cut into approximately 1-cm lengths. The epithelial cell layer was removed by vigorous stirring in fluorescence activated cell sorting (FACS) buffer (PBS containing 3% FCS, 20 mM HEPES, 10 μg/ml polymyxin B, 100 U/ml penicillin, 100 μg/ml streptomycin, 1 mM sodium pyruvate, and 10 mM EDTA) for 20 min at 37°C. After intensive washing with PBS, the intestinal fragments were minced, and then digested in 400 U/ml collagenase D and 10 μg/ml DNase I (Roche, Mannheim, Germany) at 37°C for 45 min with continuous stirring. EDTA was then added (500 mM final concentration), and the cell suspension was incubated for an additional 5 min at 37°C, filtered through a 100-μm cell strainer, and subjected to 40%/75% Percoll (Amersham Biosciences) density-gradient centrifugation. Cells at the interface were collected, washed, and used for phenotypic analysis and intracellular cytokine staining.

MLNs and spleens were disrupted with a syringe pump in RPMI 1640 medium, supplemented with 10% FCS, 100 U/mL penicillin, 100 μg/mL streptomycin, 10 mM HEPES, and 11 mM sodium carbonate. The crushed spleens were lysed with ACK lysis buffer (0.15 M NH_4_Cl, 10 mM KHCO_3_, 0.1 mM Na _2_EDTA [pH 7.3]), subjected to enzymatic digestion, and the obtained cells were used directly for phenotypic analysis.

### Antibodies and Flow cytometry

To assess the expression of surface markers on T and B lymphocytes, cells were stained with Pacific Blue (PB)-conjugated anti-CD4 (RM4-5), fluorescein isothiocyanate (FITC)-conjugated anti-CD8α (53-6.7), allophycocyanin-conjugated anti-T cell antigen receptor (TCR) β chain (H57-597), and phycoerythrin (PE)-cyanine (Cy) 7-conjugated anti-CD19 (1D3), after FcR blocking by anti-mouse CD16/CD32 (2.4G2; all from BD Biosciences, San Jose, CA, USA). To identify the regulatory T cells, T cells were stained with PB-conjugated anti-CD4. The surface staining was followed by permeabilization with Foxp3 fix/perm solution (eBioscience, San Diego, CA, USA) and intracellular staining with a PE-conjugated anti-Foxp3 antibody (FJK-16s, eBioscience), according to the manufacturer’s protocol.

For the SI LP APCs, the antibodies used were PE-Cy7-conjugated anti-CD11b (M1/70; BD Biosciences), allophycocyanin-conjugated anti-CD11c (HL3; BD Biosciences), and PB-conjugated anti-major histocompatibility complex (MHC) class II (M5/114.15.2; eBioscience). For eosinophil identification, the antibodies used were PE-Cy7-conjugated anti-CD11b, FITC-conjugated anti-CD11c (N418; eBioscience), and eFluor® 450-conjugated anti-MHC class II (M5/114.15.2; eBioscience), PE-conjugated anti-sialic acid-binding Ig-like lectin F (Siglec F; E50/2440; BD Biosciences), and Alexa Fluor® 647-conjugated anti-CD193 (CCR3) (83103; BD Biosciences). The expression of costimulatory molecules was determined using the following mAbs: FITC-conjugated anti-CD80 (16-10A1), PE-conjugated anti-CD86 (GL1), and PE-conjugated anti-CD40 (1C10; all from eBioscience). FITC-conjugated anti-American hamster Immunoglobulin G and PE-conjugated anti-rat IgG2a K, used as isotype controls, were from eBioscience.

T lymphocytes isolated from the SI LP, MLNs, and spleen were stimulated with 50 ng/mL phorbol 12-myristate 13-acetate (PMA) and 750 nM ionomycin (both from Sigma-Aldrich, St. Louis, MO, USA), to which 1 µL GolgiStop (BD Pharmingen) was added, for 4 h at 37°C and 5% CO_2_. The stimulated cells were resuspended in FACS buffer. After the Fc receptors were blocked with anti-mouse CD16/CD32 (2.4G2) for 15 min at 4°C, the cells were stained with anti-CD4 for 30 min at 4°C. To examine the intracellular cytokine expression, the cells were fixed and permeabilized using the Cytofix/Cytoperm Kit (BD Pharmingen) and then stained with anti-interferon (IFN)-γ (XMG1.2; eBioscience), anti-IL-17A (JES5-16E3; BD Biosciences), and anti-IL-10 (JESS-16E3; BD Biosciences). Flow cytometric analysis was performed on a Gallios (Beckman Coulter, Miami, FL, USA), and the data were processed with FlowJo software (Tree Star, San Carlos, CA, USA).

### Measurement of UDE-specific IgE, IgG1, and total IgE levels in sera from UDE- and PBS-administered mice

Serum UDE-specific IgE, IgG1, and total IgE antibody levels were evaluated using enzyme-linked immunosorbent assay (ELISA). For determination of UDE-specific IgG1 and IgE levels, 10 μg/mL UDE was coated onto 96-well, flat bottom plates and incubated overnight at 4°C. The plate was washed twice with wash buffer (PBS, 0.05% Tween-20) and then blocked with blocking buffer (PBS, 0.05% Tween-20, 5% skimmed milk) for 1h at room temperature (RT). The plate was washed twice with wash buffer and sera from 50mg/mL UDE- or PBS-administered mice were added, and incubated overnight at 4°C. After the plate was washed 5 times with wash buffer, horseradish peroxidase (HRP)-conjugated anti mouse IgE or IgG1 (Southern Biotech, AL, USA) diluted in blocking buffer (1:3000) was added to each well. After the incubation for 1.5h at RT, the plate was washed 7 times with wash buffer and 3,3',5,5'-tetramethylbenzidine (TMB) was added to each well. The reaction was stopped with 2N H_2_SO_4_, and the optical density of the samples at 450 nm was determined by a Versamax microplate reader (Molecular Devices, Sunnyvale, CA, USA). For total IgE measurement, a commercial Mouse IgE ELISA Quantitation Set (Bethyl Laboratory, Inc., TX, USA) was used according to the manufacturer’s instructions.

### Statistics

Statistical analysis was conducted with GraphPad Prism 5 software. Two-group comparisons were performed using an unpaired Student’s *t*-test. *p* values < 0.05 were considered statistically significant. Error bars denote ± SEM.

## Results

### CD4^+^ T cells in the SI LP were reduced by UDE administration

**Figure 1:**
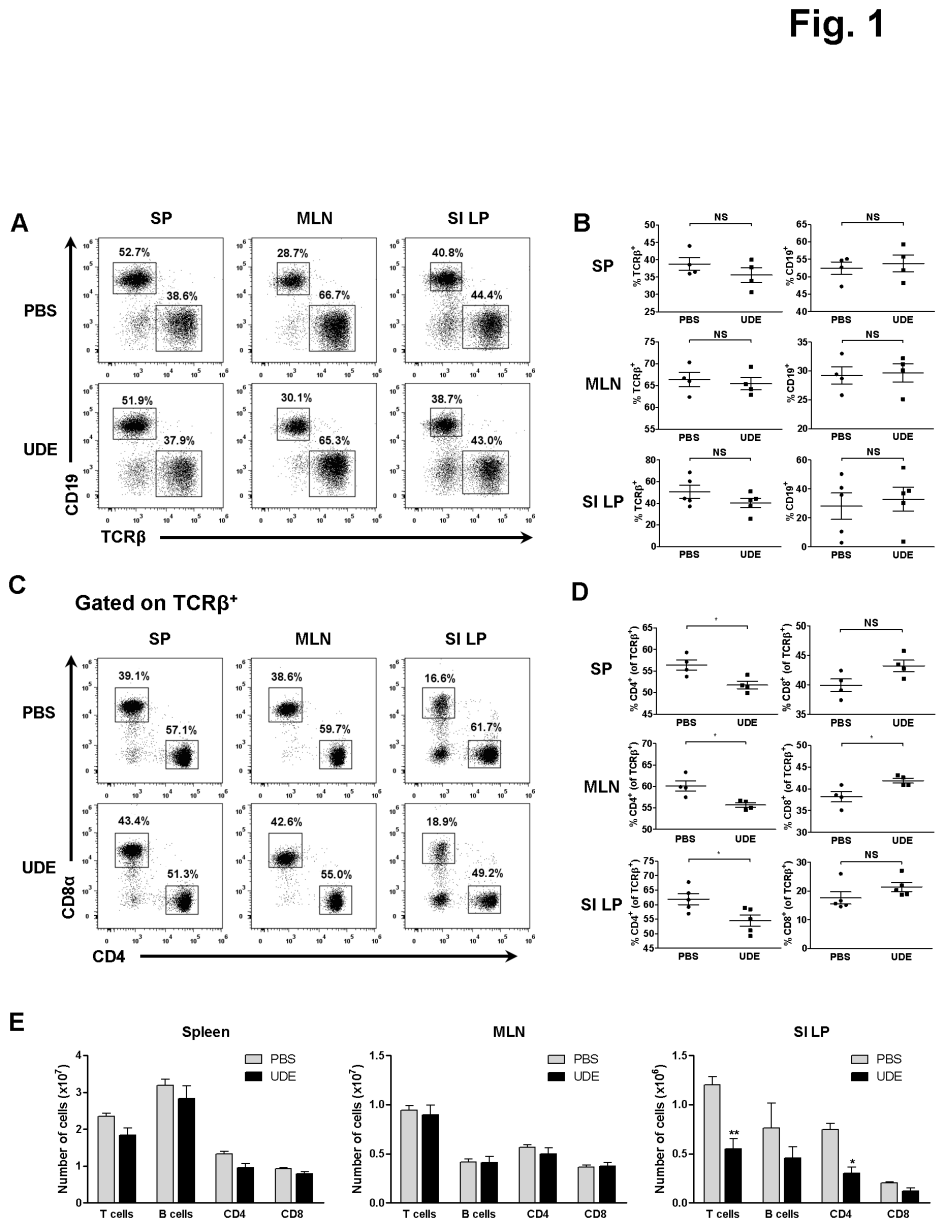
Decreased number of CD4^+^ T cells in the SI LP of UDE-administered mice. (A) Cells isolated from spleen (SP), mesenteric lymph node (MLN), and small intestinal lamina propria (SI LP) were stained with TCRβ and CD19 and analyzed by flow cytometry. (B) Frequency of T and B cells in the SP, MLN, and SI LP of UDE- versus PBS-administered control mice. (C) T Cells from SP, MLN, and SI LP were stained with CD4 and CD8α and analyzed by flow cytometry. (D) Frequency of CD4 and CD8 cells in the SP, MLN, and SI LP of UDE- versus PBS-administered control mice. (E) Total number of T/B cells and CD4/CD8 cells in the SP, MLN, and SI LP of UDE- versus PBS administered control mice. *p < 0.05, **p < 0.01, NS=not significant.

To determine whether UDE has an effect on lymphocytes, we first analyzed the number and percentage of cells that expressed the surface marker TCR β chain and CD19, the hallmark of T and B lymphocytes, respectively. As shown in Figure 1E, UDE noticeably suppressed the number of T cells in the SI LP without affecting other secondary lymphoid tissues (the spleen and MLNs). In contrast, UDE did not significantly influence the B cell populations of the spleen, MLNs, or SI LP (Figure 1A, B, and E). Among the T cells, the number of CD4^+^ T cells was significantly reduced only in the SI LP of UDE-treated mice compared with PBS-administered control mice despite the slight but significant reduction of percentage of CD4^+^ T cells in SP and MLN. The number of CD8^+^ T cells was unchanged in the spleen, MLNs, and SI LP (Figure 1E).

### Th1 and Th17 cells in the SI LP were decreased by UDE administration

**Figure 2:**
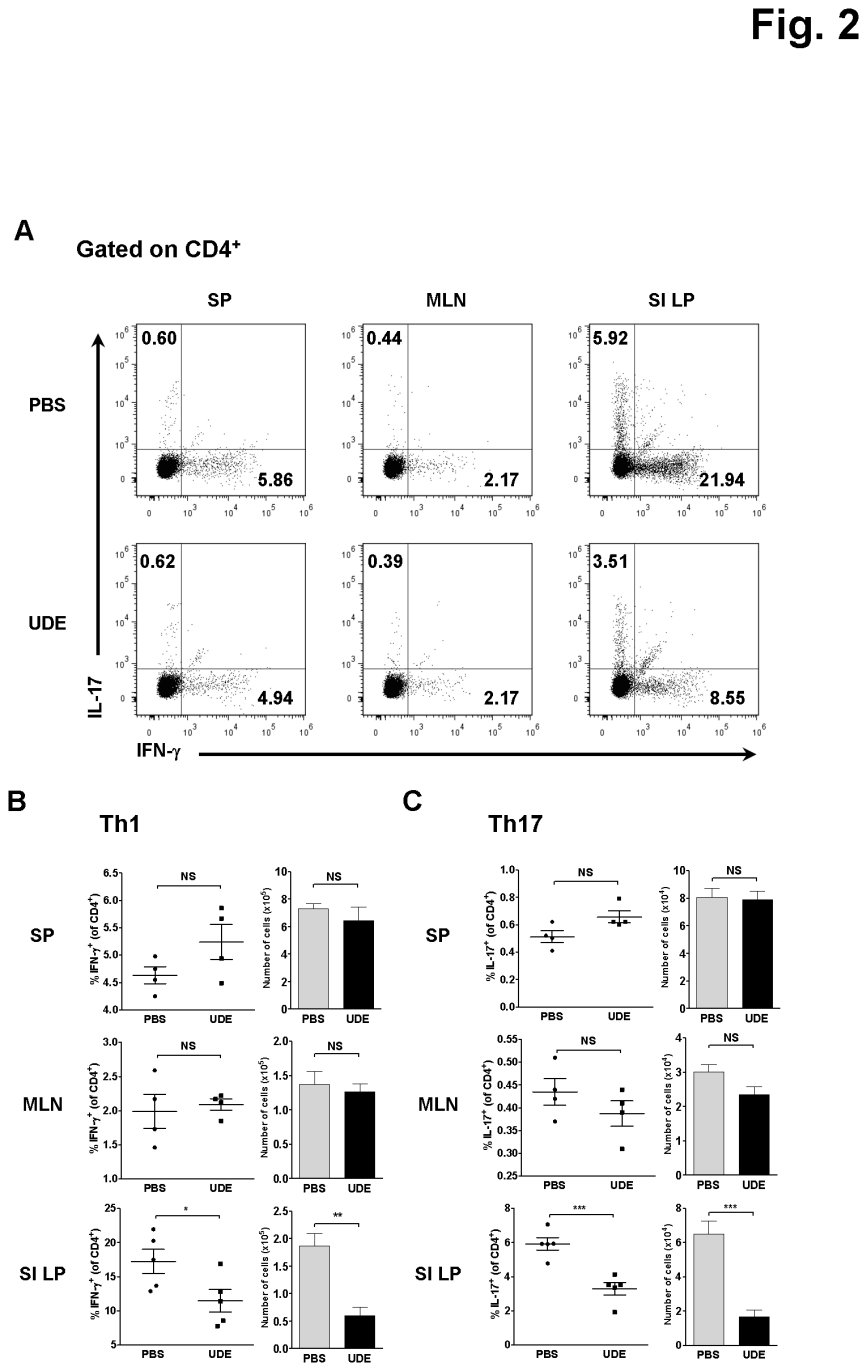
Decreased number and frequency of Th1 and Th17 cells in the SI LP of UDE-administered mice. (A) Intracellular cytokine staining for IL-17 and IFN-γ in CD4^+^ T cells in SP, MLN, SI LP from UDE- versus PBS-administered control mice. Cells were stimulated with PMA and Ionomycin for 4 hours and analyzed by flow cytometry. (B) Frequency and number of Th1 cells in the SP, MLN, and SI LP of UDE- versus PBS-administered control mice. (C) Frequency and number of Th17 cells in the SP, MLN, and SI LP of UDE- versus PBS-administered control mice. *p < 0.05, **p < 0.01, ***p < 0.001, NS= not significant.

Next, to investigate whether UDE affects the differentiation of T helper cell subsets, we assessed the number and percentage of T helper cells that produce IFN-γ and IL-17, the signature cytokines of Th1 and Th17 cells, respectively. Notably, the number and percentage of both Th1 and Th17 cells were significantly decreased in the SI LP of UDE-treated mice, but those in the other tissues were not affected (Figure 2).

**Figure 3:**
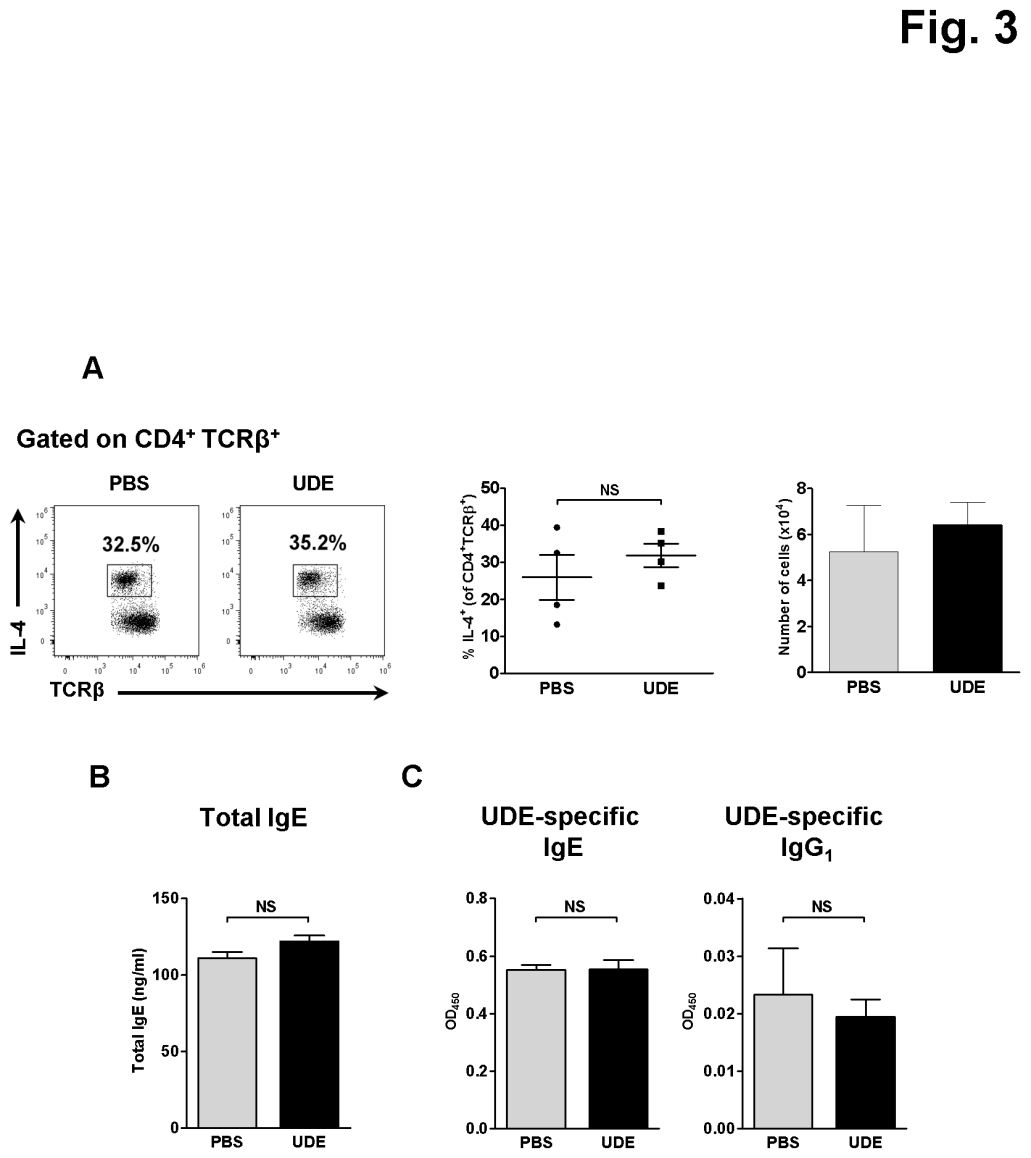
No significant differences of Th2 cells in the SI LP and serum total IgE, UDE-specific IgE and IgG1 levels between UDE- and PBS-administered mice. (A) Frequency and number of IL-4/GFP^+^ CD4^+^ T cells in the SI LP of UDE-versus PBS-administered control 4get mice. (B) Serum total IgE, UDE-specific IgE, and IgG1 secretion analyzed by ELISA. NS= not significant, OD_450_=optical density at absorbance 450nm.

It is well known that IL-4, the signature cytokine of Th2 cells, can negatively regulate the development of both Th1 and Th17 subsets [13]. Thus, we tested if UDE administration antagonizes the Th1 and Th17 development by raising the IL-4 production in SI LP using the 4get mice [9]. As shown in Figure 3A, there were no significant differences in the number and percentage of the IL-4-producing CD4^+^ T cells between the UDE- and PBS-administered 4get mice. We also tested if the mice treated with UDE develop UDE-specific IgG1 or IgE antibodies. Serum levels of UDE-specific IgG1 and IgE as well as the total IgE were not changed in UDE-administered mice compared with PBS control mice (Figure 3B and C). The results demonstrate that oral UDE administration neither induces the UDE-specific antibodies nor influences the Th2 activities.

### Treg activity did not induce down-regulation of the Th1 and Th17 responses

**Figure 4:**
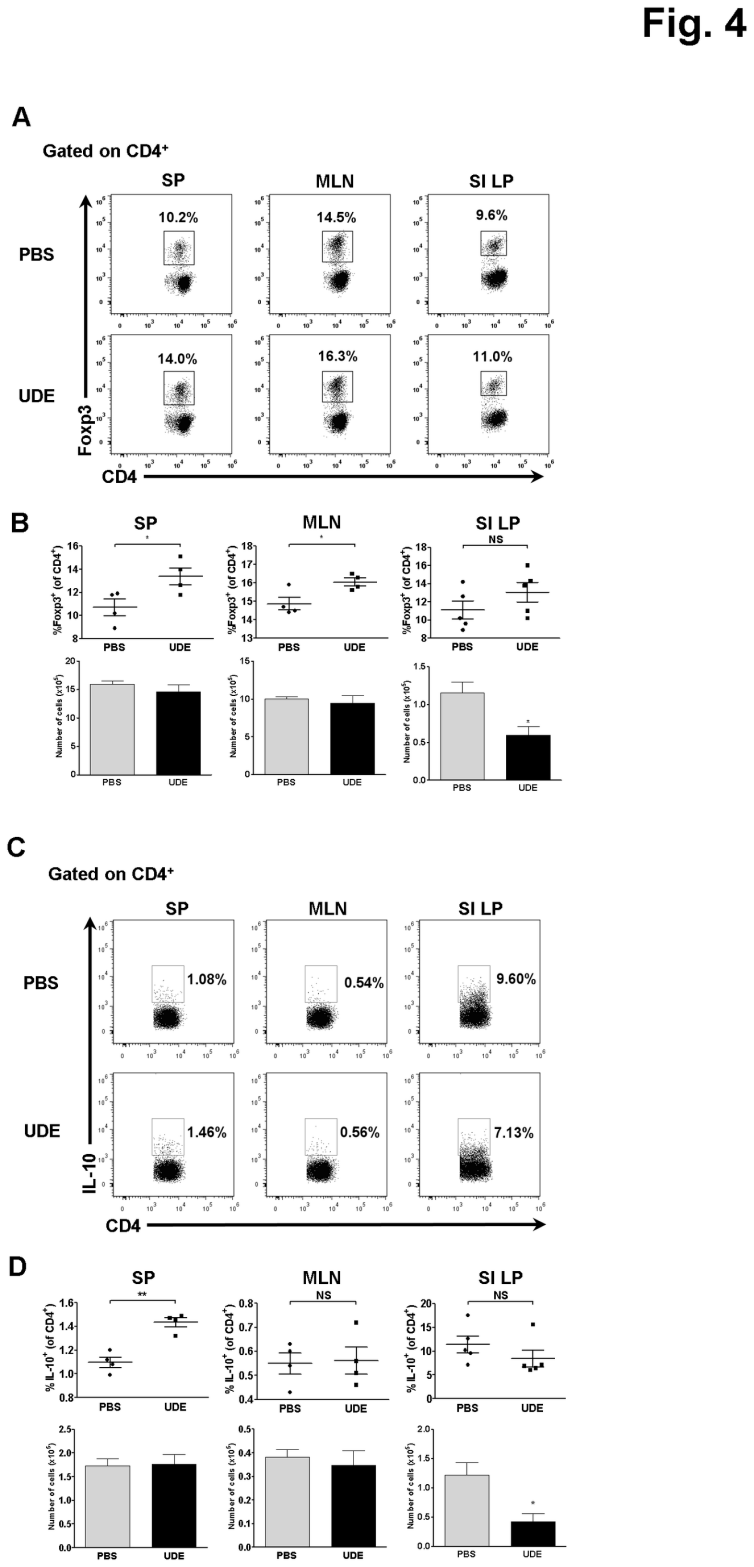
No significant increase of CD4^+^ Foxp3^+^ regulatory T cells and IL-10-producing CD4^+^ T cells in the SI LP of UDE-administered mice. (A) Intracellular staining of Foxp3 in CD4^+^ T cells from UDE- versus PBS-administered control mice. (B) Frequency and number of CD4^+^ Foxp3^+^ regulatory T cells in the SP, MLN, and SI LP of UDE-versus PBS-administered control mice. (C) Intracellular cytokine staining for IL-10 in CD4^+^ T cells from UDE- and PBS-administered control mice. (D) Frequency and number of IL-10-producing T helper cells in the SP, MLN, and SI LP of UDE- versus PBS-administered control mice. *p < 0.05, **p < 0.01, NS=not significant.

To determine whether the down-regulation of the Th1 and Th17 populations in the SI LP of UDE-treated mice was caused by a reciprocal skewing toward Tregs, we investigated the alteration in the number and frequency of Tregs. The percentage but not the total cell number of CD4^+^ Foxp3^+^ Tregs was slightly increased in the MLNs and spleen. In contrast, the number of CD4^+^ Foxp3^+^ Tregs was significantly lower in the SI LP from the UDE-treated mice while the percentage of CD4^+^ Foxp3^+^ Tregs in the SI LP did not change (Figure 4A and B). Similarly, the number but not the percentage of IL-10-producing CD4^+^ cells (Tr1) in the SI LP was significantly decreased in the UDE-treated mice (Figure 4C and D). Taken together, these results indicate that down-regulation of the Th1 and Th17 subsets in the SI LP of UDE-treated mice did not result from the reciprocal up-regulation of regulatory T cell activities and suggest that another cell type might be responsible for the UDE-induced down-regulation of the Th1 and Th17 cell differentiation.

### The number and proportion of LP eosinophils was increased by UDE administration

We next examined whether UDE influenced APCs such as DCs, macrophages, and eosinophils in the SI LP. Intestinal APCs can be distinguished on the basis of their CD11b and CD11c expression [1]. In brief, the CD11c^high^ CD11b^low^ and the CD11c^high^ CD11b^high^ subsets correspond to CD8α^int^ and CD8α^-^ DCs, respectively, whereas the CD11b^high^ CD11c^int^ and the CD11b^int^ CD11c^int^ cell subpopulations are eosinophils and macrophages, respectively. In addition, LP eosinophils express no or a negligible amount of MHC class II (MHC II), whereas DCs and macrophages express relatively high levels of MHC II [1]. Therefore, in the current study, LP eosinophils were quantified by flow cytometry by gating on the cells with low MHC II.

**Figure 5:**
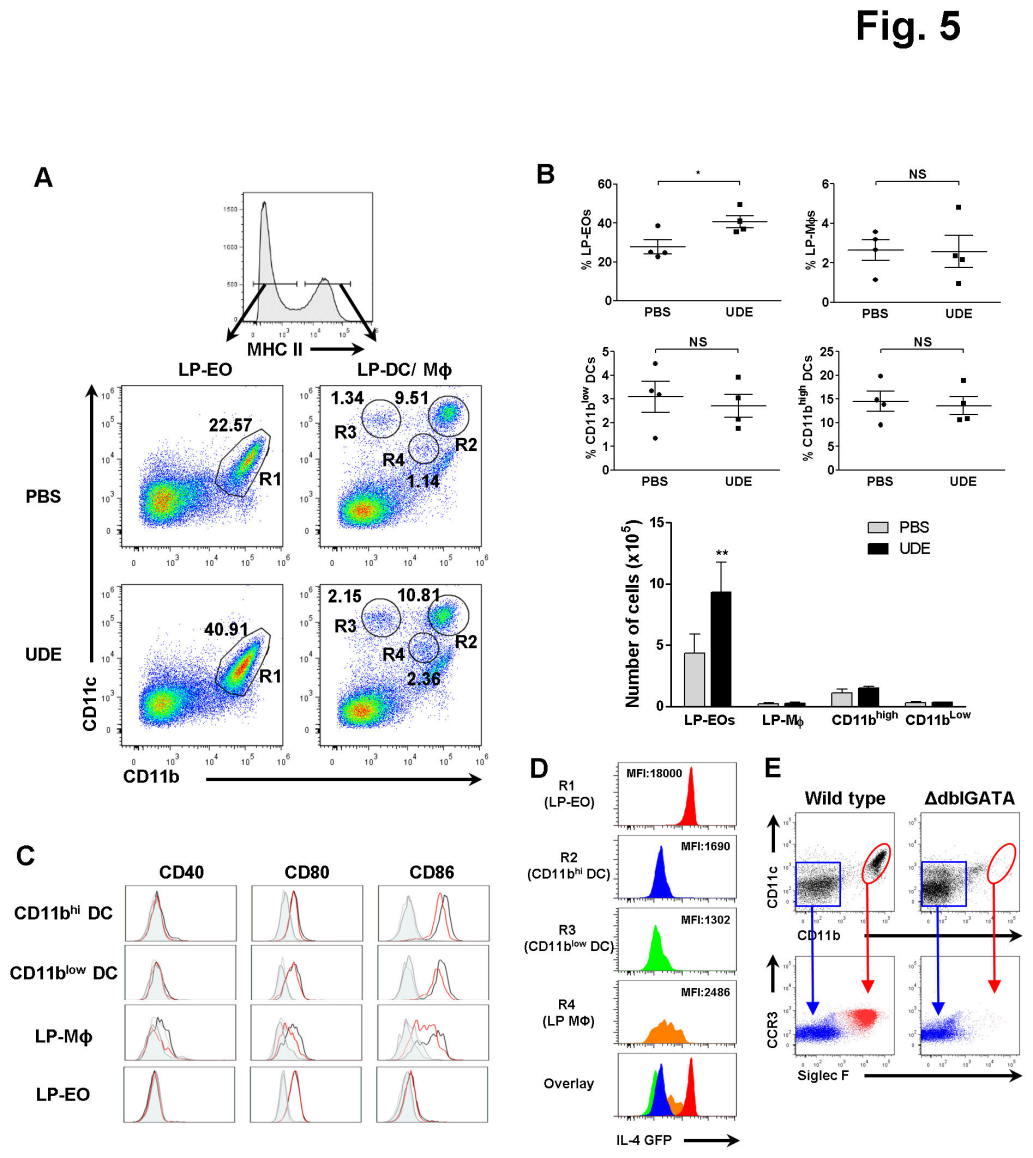
Significant increase of IL-4-expressing eosinophils in the SI LP of UDE-administered mice. (A) Cells isolated from SI LP were stained with CD11b, CD11c, and MHC class II and analyzed by flow cytometry. MHC class II ^low^CD11b^high^ CD11c^int^ cells (R1) are eosinophils. Among MHC class II^high^ cells, CD11b ^int^CD11c^int^ cells (R4) and CD11c^high^ cells (R2 and R3) correspond to macrophages and dendritic cells, respectively. (B) Proportion and number of eosinophils, macrophages, and dendritic cells in the SP of UDE- versus PBS-administered control mice. (C) Antigen presenting cells isolated from SI LP were stained with CD40, CD80, and CD86 and analyzed by flow cytometry. Empty, solid line histograms represent cells from UDE-administered (red) versus PBS-administered control mice (black). Shaded histograms represent cells stained with isotype control of UDE-administered (dark gray) versus PBS-administered control mice (gray). (D) Mean fluorescence intensity of IL-4/GFP expressed by APCs from the SI LP of 4get mice were analyzed by flow cytometry. (E) Analysis of CD11b^high^CD11c^int^ cells from LP of WT and ΔdblGATA mice for eosinophil markers CCR3 and Siglec F. *p<0.05, **p < 0.01, NS= not significant, LP: lamina propria, EO: eosionophil, DC: dendritic cell, Mϕ: macrophage, MFI: mean fluorescence intensity.

Interestingly, the number and frequency of LP eosinophils from UDE-treated mice were markedly increased compared with the PBS controls, while there were no significant differences in the DCs or macrophages (Figure 5A and B). For the costimulatory molecules, the DCs and macrophages from the UDE-treated mice showed slightly decreased expressions compared with controls. However, there was no difference of costimulatory molecule expression in the eosinophils, which expressed a higher level of CD80 relative to the levels of CD86 and CD40 (Figure 5C).

Next, to assess the IL-4 expression in the intestinal APCs, we analyzed the mean fluorescence intensity (MFI) of GFP in the intestinal APCs of 4get mice. The GFP expression levels in LP eosinophils (R1) were 7- to 13-fold higher than those in other LP APCs (R2, R3, and R4), suggesting that the main source of IL-4 is CD11b^hi^ CD11c^int^ eosinophils in the gut (Figure 5D). To verify that the CD11b^hi^CD11c^int^ SI LP cells are indeed eosinophils, we stained LP cells from wild type (WT) and eosinophil-deficient ΔdblGATA mice for expression of the eosinophil markers CCR3 and Siglec F. ΔdblGATA mice are selectively deficient in eosinophils due to deletion of the high-affinity GATA-binding site in the GATA-1 promoter [10]. As expected, in WT mice the CD11b^high^CD11c^int^ LP cells were double positive for CCR3 and Siglec F staining, confirming their identity as eosinophils. In contrast, both CCR3^+^ Siglec F^+^ and CD11b^high^CD11c^int^ LP cell populations were not observed in ΔdblGATA mice, demonstrating that CD11b^hi^CD11c^int^ SI LP cells unequivocally correspond to eosinophils (Figure 5E). These data indicate that UDE up-regulates the total number and frequency of SI LP eosinophils, which can down-regulate the Th1 and Th17 responses via IL-4 secretion and contribute to regulate intestinal inflammation.

## Discussion

Dysregulation of intestinal immune homeostasis, the balanced state between the robust immunity required for host defense and the immune tolerance toward self and dietary antigens, can result in chronic inflammation, such as inflammatory bowel disease (IBD). IBD is a chronic relapsing inflammatory disorder, characterized by CD4^+^ T lymphocyte and neutrophil infiltration into the alimentary tract. IBD has two main subtypes, Crohn’s disease (CD) and ulcerative colitis (UC), which are classified according to the inflamed sites and clinical manifestations. Among the various etiologies of IBD, the most fundamental are genetic factors. Individuals with mutations in Caspase Recruitment Domain 15 (CARD15)/Nucleotide Oligomerization Domain 2 (NOD2) are more susceptible to IBD, due to aberrant pro-inflammatory immune responses triggered by hyperreactivity to commensal microbiota. This destructive cytokine response is mainly directed by abnormal T cell differentiation patterns [14]. Murine experimental colitis models have shown that CD4^+^ T cells play an important role in the pathogenesis of IBD by producing pro-inflammatory cytokines [15]. CD is believed to be associated with Th1/Th17-mediated responses induced by excessive IL-12 and IL-23 production whereas UC is linked to an inappropriate increase in the production of Th2 cytokines such as IL-5 and IL-13 [16].

Th1 cells belong to one of the principal T helper cell subsets and produce IFN-γ to clear deleterious intracellular microorganisms. Th17 subset is a more recently described pro-inflammatory T helper cell lineage and produces IL-17 and IL-22 to eliminate specific types of pathogens that require a massive inflammatory response [17]. Th17 cells are constitutively present in the SI LP and cooperate with Th1 cells to provide protective immunity against pathogens in the gut. However, an exaggerated Th17 response can give rise to chronic inflammation and autoimmune disorders. For this reason, pro-inflammatory T helper cell responses need to be controlled by regulatory mediators.

Regulatory T cells play a critical role in the induction and maintenance of immune tolerance, contributing to the immune homeostasis of the gut. Dysfunction of Treg activity is implicated in uncontrolled inflammatory disorders such as IBD. Several studies showed that patients with IBD have a much lower frequency of Foxp3^+^ CD4^+^ Tregs than healthy individuals and that the level of IL-10, a major cytokine produced by Treg, is also decreased in the patients’ intestinal mucosa [18,19]. Maintaining an intact homeostatic environment in the gut requires a balance between the pro-inflammatory Th1/Th17 cell responses and the anti-inflammatory Treg cell activities. The present study indicated that UDE administration markedly attenuated the Th1 and Th17 activities (Figure 2). But, interestingly the attenuation was not due to a significant increase in CD4^+^ Foxp3^+^ Tregs (Figure 4A and B) or elevated IL-10 production (Figure 4C and D).

APCs in the SI LP are also crucial tuners of the intestinal homeostasis. LP DCs determine the critical steps in controlling the immunity against pathogens and tolerance toward commensals. LP DCs can drive naïve CD4^+^ T cells to differentiate into CD4^+^ Foxp3^+^ Tregs in a transforming growth factor β- and retinoic acid-dependent manner [20] and perform a tolerogenic function by constitutively migrating through the lymph to the draining MLNs, where they present antigen to T cells [2]. In addition, LP macrophages may have a fundamental role in maintaining mucosal tolerance by their expression of immunomodulatory molecules, hyporesponsiveness to Toll-like receptor stimulation, spontaneous production of large amounts of IL-10, and ability to suppress the differentiation of Th1 and Th17 cells while promoting the Treg differentiation [4].

Besides these professional APCs, eosinophils are known to have an antigen-presenting function. Previous studies showed that eosinophils express costimulatory molecules such as CD40, CD80, and CD86 and present antigen to T cells [21]. As shown in Figure 5A and B, UDE-treated mice demonstrated a substantial increase in SI LP eosinophils compared with the PBS-administered control mice, without an effect on the macrophages or DCs. However, there was no apparent alteration of the costimulatory molecules in eosinophils (Figure 5C), which implies that UDE’s contribution to immune regulation in the gut does not involve the induction of eosinophils into a tolerogenic APC.

Most studies on eosinophils have focused on their properties as effector cells under pathological conditions, such as parasitic infections and allergic diseases. Eosinophils are well known to accumulate in the intestinal mucosa during parasitic infections and intestinal inflammatory disorders such as immunoglobulin E-mediated food allergy, eosinophilic gastroenteritis, and IBD [22]. However, recently increasing attention has been paid to the physiological roles of tissue-resident eosinophils under steady-state conditions. Notably, a considerable number of steady-state eosinophils are found to be constitutively present in the SI LP. A recent study demonstrated that intestinal eosinophils express high levels of the pan B cell marker, CD22, which negatively regulates tissue eosinophilia [23]. According to another study, the survival of intestinal eosinophils depends on common γ chain signaling and intestinal eosinophils have a more prolonged turnover rate than their pulmonary or circulating counterparts [24]. These properties suggest that the intestinal eosinophils have special functions distinct from the eosinophils dwelling in other tissues, such as the lungs and blood. Intestinal eosinophils are thought to interact with adjacent T lymphocytes in the SI LP and to play a critical role in regulating the mucosal immune response under steady-state conditions [25].

Through various studies, it has already been investigated that the stem and root bark of *Ulmus davidiana* var. *japonica* Nakai (UD) extracts or its compounds have anti-inflammatory activity [7,26-29], but the influence of UD on steady-state eosinophils in the GI tract has not been definitely elucidated so far. According to our unpublished data, the frequencies of Th1 and Th17 were significantly increased in the SI LP of ΔdblGATA mice compared with WT mice. This observation is consistent with our data from the current study, suggesting that the elevated level of steady-state SI LP eosinophils caused by UDE administration induces the suppression of the Th1 and Th17 responses. From these findings, we speculate that the SI LP eosinophils play a major role in intestinal homeostasis as a prominent modulator of the immune responses. The UDE-induced increase in steady-state eosinophils in the SI LP is different from the pathological accumulation of eosinophils observed in IBD or eosinophilic gastroenteritis. It appears that under physiological conditions, tissue eosinophilia contributes to baseline homeostasis by regulating immune activities [30].

In summary, our data demonstrate that UDE can increase the number and frequency of LP eosinophils and downregulate the Th1 and Th17 cell responses. UDE is a promising therapeutic candidate for the treatment of chronic intestinal inflammatory conditions such as Crohn’s disease, which caused by excessive pro-inflammatory T helper cell activities.
